# Supplementary material for: Immunogenicity, Immune Dynamics, and Subsequent Response to the Booster Dose of Heterologous versus Homologous Prime-Boost Regimens with Adenoviral Vector and mRNA SARS-CoV-2 Vaccine among Liver Transplant Recipients: A Prospective Study
Source: Vaccines (Basel). 2022 Dec 12;10(12):2126. doi: 10.3390/vaccines10122126 (PMC9781301; doi:10.3390/vaccines10122126)

Supplementary Table S1. Baseline characteristics of LT recipients evaluated for SARS-CoV-2 specific T-cell response

**Parameter**	**ChAdOx1/BNT162b2 (n=41)**	**ChAdOx1/ ChAdOx1 (n=17)**	***p*-value**
Age (years)	58.9 ± 12.2	56.9 ± 16.6	0.66
Sex, male (%)	15 (36.6)	3 (17.6)	0.22
BMI (kg/m^2)	24.7± 3.3	24.8 ± 5.1	0.92
Time after transplantation (years)*	5.7 (2.9-12.4)	5.1 (2.7-10.3)	0.56
Comorbidity (%)			
HT	20 (48.8)	4 (23.5)	0.09
DM	11 (26.8)	8 (47.1)	0.22
DLP	19 (46.2)	7 (41.2)	0.78
CKD	5 (12.2)	4 (23.5)	0.43
Tacrolimus (%)	27 (65.9)	13 (76.5)	0.54
Cyclosporine (%)	5 (12.2)	3 (17.6)	0.68
Mycophenolate mofetil (%)	26 (63.4)	6 (35.3)	0.81
Sirolimus (%)	8 (19.5)	4 (23.5)	0.73
Everolimus (%)	8 (19.5)	2 (11.8)	0.71
Prednisolone	2 (4.9)	2 (11.8)	0.56
TB (mg/dl)	0.7 ± 0.4	0.7 ± 0.2	0.98
DB (mg/dl)	0.3 ± 0.1	0.3 ± 0.1	0.59
AST (U/L)	23.0 ± 8.5	24.3 ± 5.1	0.44
ALT (U/L)	24.7 ± 19.7	24.8 ± 5.1	0.97
ALP (U/L)	84.7 ± 51.0	72.3 ± 27.6	0.24
Albumin (g/dl)	4.2 ± 0.3	4.3 ± 0.3	0.81
Hemoglobin (g/dl)	14.0 ± 3.7	14.0 ± 1.6	0.40
White blood cell (10^3/ul)	6.1 ± 2.0	5.5 ± 2.0	0.35
Platelet (10^3/ul)	225.1 ± 90.3	197.0 ± 82.4	0.26

Supplementary Table S2. Adverse events after SARS-CoV-2 vaccination in LT recipients

Adverse events	ChAdOx1/BNT162b2 (n=64)	ChAdOx1/ChAdOx1 (n=25)	*p*-value
**First vaccination**			
Pain at injection site	31.3%	24.0%	0.61
Fever	20.3%	28.0%	0.62
Headache	10.9%	8.0%	0.68
Myalgia	6.3%	12.0%	0.40
Nausea	3.1%	12.0%	0.12
Diarrhea	4.7%	0.0%	0.56
Others	2.2%	2.0%	0.84
**Second vaccination**			
Pain at injection site	32.8%	24.0%	0.27
Fever	9.4%	8.0%	0.84
Headache	4.7%	8.0%	0.54
Myalgia	3.1%	4.2%	0.83
Nausea	7.8%	4.0%	0.52
Diarrhea	1.6%	0.0%	0.53
Others	0.9%	1.2%	0.72
**Booster vaccination**			
Pain at injection site	29.7%	24.0%	0.63
Fever	6.3%	12.0%	0.40
Headache	7.8%	8.0%	0.97
Myalgia	1.6%	8.0%	0.06
Nausea	4.7%	4.7%	0.89
Diarrhea	1.6%	4.0%	0.49
Others	0.8%	0.7%	0.66

Supplementary Figure S1. Flowchart of study cohort

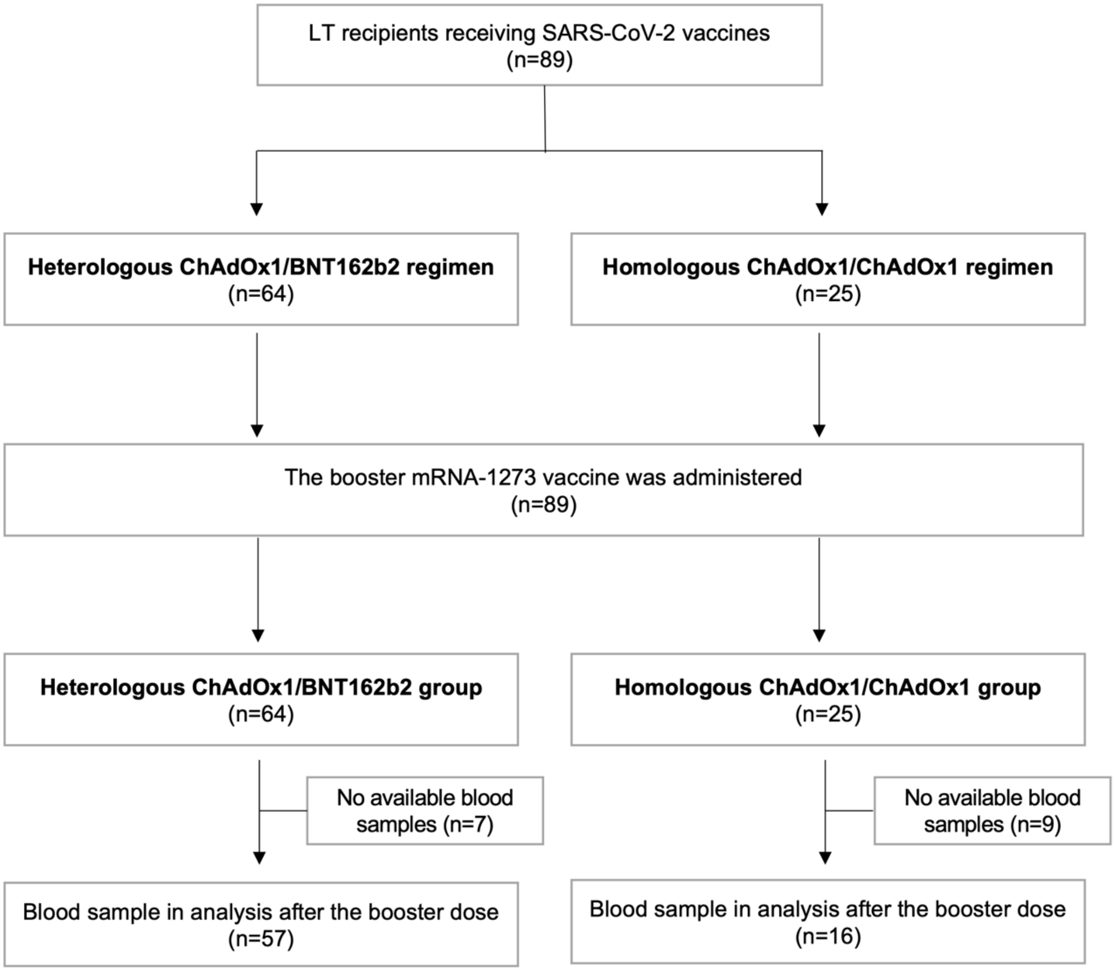

Supplement: Supplementary file 1 [file vaccines-10-02126-s001.zip › vaccines-2072876-supplementary.pdf]
